# *N*,*N*-Dihexyl-4-[2-(4-nitro­phen­yl)vin­yl]aniline

**DOI:** 10.1107/S1600536811016709

**Published:** 2011-05-11

**Authors:** Dieter Schollmeyer, Heiner Detert

**Affiliations:** a University Mainz, Duesbergweg 10-14, 55099 Mainz, Germany

## Abstract

The title compound, C_26_H_36_N_2_O_2_, was prepared by Horner olefination of *p*-dihexyl­amino­benzaldehyde and diethyl *p*-nitro­benzyl­phospho­nate. It crystallizes with two independent mol­ecules in the asymmetric unit. Both have similar geometries of the π-systems but the conformations of all hexyl chains are different. Whereas one hexyl chain of the first mol­ecule shows the typical all-*anti* conformation, the second is arranged in a *gauche*-*anti*-*gauche*-anti conformation with N—C—C—C, C—C—C—C, C—C—C—C and C—C—C—C torsion angles of −65.1 (4), 167.3 (3), 63.3 (4), and 179.4 (3)°. One of the hexyl chains in the other mol­ecule has an *anti*-*anti*-*gauche*-*anti* conformation [N—C—C—C, C—C—C—C, C—C—C—C and C—C—C—C torsion angles = 179.6 (3), −179.8 (3), −68.7 (5) and −178.8 (4)°], the other starts with an *anti*-*gauche*-*gauche* sequence. Molecules *A* and *B* are composed of five planar subunits. The angle sums around the N atoms are in the range 356 (2)–360.0 (2)°. Torsion angles between these segments do not exceed 4.9 (4)°, except for one of the alkyl chains each [molecule *A* = 26.2 (4)°; molecule *B* =  −6.0 (4)°]. The high planarity of the molecules and the short aniline C—N bonds [1.385 (3) Å in molecule *A* and 1.378 (3) Å in molecule *B*] indicate a strong electronic coupling through the stilbene unit. One methylene group is disordered over two positions with an occupancy ratio of 0.72:0.28.

## Related literature

For chromophores and fluoro­phores based on quadrupolar donor–acceptor-substituted stilbenoid systems, see: Detert & Sugiono (2005 ▶); Strehmel *et al.* (2003 ▶); Nemkovich *et al.* (2010 ▶). Similar amino­nitro­stilbenes had been prepared earlier, see: Pfeiffer *et al.* (1915 ▶); Chardonnens & Heinrich (1939 ▶); Meier *et al.* (2004 ▶). The optical properties of these dyes are strongly dependent on charge transfer and torsion angles, see: Baumann *et al.* (1977 ▶); Goerner (1998 ▶); Dekhtyar & Rettig (2007 ▶). Conjugated oligomers with basic sites are sensing materials for polarity and cations, see: Wilson & Bunz (2005 ▶); Zucchero *et al.* (2009 ▶). For a comparable compound, see: Fischer *et al.* (2011 ▶).
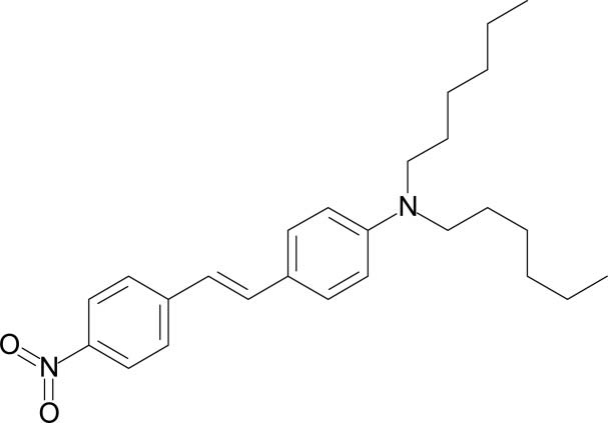


## Experimental

### 

#### Crystal data

C_26_H_36_N_2_O_2_
*M* *_r_* = 408.57
Triclinic, 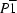

*a* = 9.6574 (9) Å
*b* = 11.4153 (10) Å
*c* = 23.604 (2) Å
α = 93.297 (3)°
β = 94.834 (3)°
γ = 112.696 (3)°
*V* = 2380.6 (4) Å^3^
*Z* = 4
Mo *K*α radiation
μ = 0.07 mm^−1^
*T* = 173 K
0.54 × 0.37 × 0.06 mm

#### Data collection

Bruker SMART APEXII diffractometer
Absorption correction: multi-scan (*PLATON*; Spek, 2009 ▶) *T*_min_ = 0.936, *T*_max_ = 0.996
90478 measured reflections
11464 independent reflections
7228 reflections with *I* > 2σ(*I*)
*R*_int_ = 0.062

#### Refinement

*R*[*F* ^2^ > 2σ(*F* ^2^)] = 0.082
*wR*(*F* ^2^) = 0.239
*S* = 1.02
11464 reflections
553 parameters
H-atom parameters constrained
Δρ_max_ = 0.76 e Å^−3^
Δρ_min_ = −0.46 e Å^−3^

### 

Data collection: *APEX2* (Bruker, 2006 ▶); cell refinement: *SAINT* (Bruker, 2006 ▶); data reduction: *SAINT*; program(s) used to solve structure: *SIR97* (Altomare *et al.*, 1999 ▶); program(s) used to refine structure: *SHELXL97* (Sheldrick, 2008 ▶); molecular graphics: *PLATON* (Spek, 2009 ▶); software used to prepare material for publication: *PLATON*.

## Supplementary Material

Crystal structure: contains datablocks I, global. DOI: 10.1107/S1600536811016709/bt5539sup1.cif

Structure factors: contains datablocks I. DOI: 10.1107/S1600536811016709/bt5539Isup2.hkl

Supplementary material file. DOI: 10.1107/S1600536811016709/bt5539Isup3.cml

Additional supplementary materials:  crystallographic information; 3D view; checkCIF report

## Figures and Tables

**Fig. 1.**
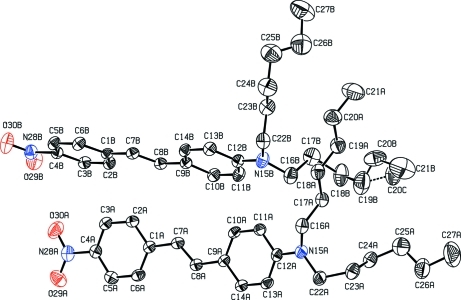
View of compound I. Displacement ellipsoids are drawn at the 50% probability level.

**Fig. 2.**
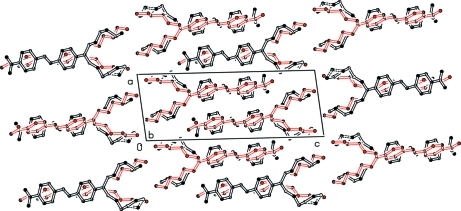
Part of the packing diagram of I. View along b-axis. Molecule A in white, molecule B in red colour.

## supplementary crystallographic information

### Comment

The title compound was prepared as a reference compound in a project focusing on chromophores and fluorophores based on quadrupolar donor-acceptor substituted stilbenoid systems, see: Detert & Sugiono (2005); Strehmel *et al.* (2003); and Nemkovich *et al.* (2010). Crystals of the title compound are composed of two independent molecules A and B with nearly identical geometries of the π-systems but different conformations of the alkyl chains. Whereas one hexyl chain of A shows the typical all-anti conformation, the second is arranged in a *gauche*-anti-*gauche*-anti conformation with torsion angles -65.1 (4)°, 167.3 (3)°, 63.3 (4)°, and 179.4 (3)°. The hexyl chains in B are also different, one has an anti-anti-*gauche*-anti conformation (torsion angles: 179.6 (3)°, -179.8 (3)°, -68.7 (5)°, and -178.8 (4)°), the other starts with an anti-*gauche*-*gauche* sequence and the penultimate C20B is disordered. The strong acceptor effect of the nitro groups through the stilbene unit is reflected by short aniline C—N-bonds: 1.385 (3)Å for C12A—N15A and 1.378 (3)Å for C12B—N15B and planar amino groups with angle sums on the aniline-N of 356° (A) and 359.7° (B). Accordingly, the stilbene framework is nearly coplanar with torsion angles of 2.7 (4)° for C7A—C8A—C9A—C10A (178.8 (2)°in B), -178.2 (2)° for C1A—C7A—C8A—C9A (179.3 (2)°in B), and -3.0 (4)° for C6A—C1A—C7A—C8A (175.9 (2)° in B). These bond lengths and torsion angles are similar to those reported for a 2,5-bis(dimethylaminostyryl)pyrazine (Fischer *et al.*, 2011). The packing of the molecules in the crystal is dominated by the voluminous side chains. Parallel but alternatingly twisted nitrostilbenes form a herringbone lattice, perpendicular to this layer, the orientation of the neighbouring molecules is antiparallel.

### Experimental

The title compound was prepared by adding potassium *tert*-butylate (1.46 g, 13 mmol) under nitrogen to a cooled solution of *p*-*N*,*N*-dihexylaminobenzaldehyde (2.17 g, 10 mmol) and diethyl *p*-nitrobenzylphosphonate (2.83 g, 10 mmol) in THF (anhyd., 50 ml) and the mixture was stirred for 2 h at 273 K and for further 2 h at ambient temperature. Acetic acid (2*M*, 5 ml) and water (70 ml) were added, the mixture was extracted with toluene (3 *x* 20 ml) and the pooled organic solutions were washed with brine (3 *x* 20 ml), dried (CaCl_2_), concentrated *in vacuo* and the title compound was isolated from the red oil by chromatography on silica gel using toluene. Red crystals with m.p. = 351 K were obtained by slow evaporation of a solution of the title compound in methanol/chloroform.

### Refinement

Hydrogen atoms attached to carbons were placed at calculated positions with C—H = 0.95 Å (aromatic) or 0.98–0.99 Å (*sp*^3^ C-atom). All H atoms were refined in the riding-model approximation with isotropic displacement parameters (set at 1.2–1.5 times of the *U*_eq_ of the parent atom). One methylene group is disordered over two positions with a site occupation factor of 0.72 for the major occupied site. For the final refinement, the site occupation factors of the disordered atoms were fixed. The highest peak (0.76 eÅ^-3^) in the final electron density map is located at 1.11Å from C16B.

### Crystal data

**Table:**

C_26_H_36_N_2_O_2_	*Z* = 4
*M**_r_* = 408.57	*F*(000) = 888
Triclinic, *P*1	*D*_x_ = 1.140 Mg m^−^^3^
Hall symbol: -P 1	Melting point: 351 K
*a* = 9.6574 (9) Å	Mo *K*α radiation, λ = 0.71073 Å
*b* = 11.4153 (10) Å	Cell parameters from 9897 reflections
*c* = 23.604 (2) Å	θ = 2.2–27°
α = 93.297 (3)°	µ = 0.07 mm^−^^1^
β = 94.834 (3)°	*T* = 173 K
γ = 112.696 (3)°	Plate, red
*V* = 2380.6 (4) Å^3^	0.54 × 0.37 × 0.06 mm

### Data collection

**Table:**

Bruker SMART APEXII diffractometer	11464 independent reflections
Radiation source: sealed Tube	7228 reflections with *I* > 2σ(*I*)
graphite	*R*_int_ = 0.062
CCD scan	θ_max_ = 28.0°, θ_min_ = 1.7°
Absorption correction: multi-scan (*PLATON*; Spek, 2009)	*h* = −12→12
*T*_min_ = 0.936, *T*_max_ = 0.996	*k* = −15→15
90478 measured reflections	*l* = −31→31

### Refinement

**Table:**

Refinement on *F*^2^	Primary atom site location: structure-invariant direct methods
Least-squares matrix: full	Secondary atom site location: difference Fourier map
*R*[*F*^2^ > 2σ(*F*^2^)] = 0.082	Hydrogen site location: inferred from neighbouring sites
*wR*(*F*^2^) = 0.239	H-atom parameters constrained
*S* = 1.02	*w* = 1/[σ^2^(*F*_o_^2^) + (0.0941*P*)^2^ + 2.795*P*] where *P* = (*F*_o_^2^ + 2*F*_c_^2^)/3
11464 reflections	(Δ/σ)_max_ < 0.001
553 parameters	Δρ_max_ = 0.76 e Å^−^^3^
0 restraints	Δρ_min_ = −0.46 e Å^−^^3^

### Special details

**Table:**

Experimental. ^1^H-NMR (CDCl_3_): δ = 8.13 ("d", J = 8.4 Hz, 2 H, 3-H, 5-H, Ph-NO_2_); 7.52 ("d", J = 8.4 Hz, 2 H, 2-H, 6-H, Ph-NO_2_); 7.39 ("d", J = 8.3 Hz, 2 H, 3-H, 5-H Ph-NHex_2_); 7.17 (d, J = 16.5 Hz, 1 H, vin); 6.87 (d, J = 16.5 Hz, 1 H, vin); 6.62 ("d", J = 8.1 Hz, 2 H, 2-H, 6-H PhNHex_2_); 3.23 ("t", 4 H, NCH_2_); 1.60 (m, 4 H, CH_2_); 1.27 (m, 12 H, CH_2_); 0.90 ("t", 6 H, CH_3_). ^13^C-NMR (CDCl_3_): δ = 148.7, 145.6, 145.2, 133.8, 128.6, 125.9, 124.1, 123.1, 120.7, 111.5, 51.1, 31.7, 27.3, 26.8, 22.7, 14.1.
Geometry. All e.s.d.'s (except the e.s.d. in the dihedral angle between two l.s. planes) are estimated using the full covariance matrix. The cell e.s.d.'s are taken into account individually in the estimation of e.s.d.'s in distances, angles and torsion angles; correlations between e.s.d.'s in cell parameters are only used when they are defined by crystal symmetry. An approximate (isotropic) treatment of cell e.s.d.'s is used for estimating e.s.d.'s involving l.s. planes.
Refinement. Refinement of *F*^2^ against ALL reflections. The weighted *R*-factor *wR* and goodness of fit *S* are based on *F*^2^, conventional *R*-factors *R* are based on *F*, with *F* set to zero for negative *F*^2^. The threshold expression of *F*^2^ > σ(*F*^2^) is used only for calculating *R*-factors(gt) *etc*. and is not relevant to the choice of reflections for refinement. *R*-factors based on *F*^2^ are statistically about twice as large as those based on *F*, and *R*- factors based on ALL data will be even larger.

### Fractional atomic coordinates and isotropic or equivalent isotropic displacement parameters (Å^2^)

**Table:**

	*x*	*y*	*z*	*U*_iso_*/*U*_eq_	Occ. (<1)
C1A	0.7433 (2)	0.6361 (2)	0.55422 (10)	0.0267 (5)	
C2A	0.6830 (3)	0.5037 (2)	0.55662 (11)	0.0292 (5)	
H2A	0.6312	0.4494	0.5230	0.035*	
C3A	0.6969 (3)	0.4498 (2)	0.60700 (11)	0.0299 (5)	
H3A	0.6565	0.3598	0.6078	0.036*	
C4A	0.7708 (3)	0.5296 (2)	0.65599 (11)	0.0286 (5)	
C5A	0.8300 (3)	0.6620 (3)	0.65598 (11)	0.0333 (5)	
H5A	0.8784	0.7154	0.6902	0.040*	
C6A	0.8169 (3)	0.7144 (2)	0.60510 (11)	0.0316 (5)	
H6A	0.8582	0.8045	0.6046	0.038*	
C7A	0.7272 (3)	0.6856 (2)	0.49919 (11)	0.0289 (5)	
H7A	0.6800	0.6240	0.4673	0.035*	
C8A	0.7720 (3)	0.8085 (2)	0.48902 (11)	0.0280 (5)	
H8A	0.8220	0.8702	0.5206	0.034*	
C9A	0.7519 (2)	0.8571 (2)	0.43441 (10)	0.0253 (5)	
C10A	0.6745 (3)	0.7776 (2)	0.38404 (11)	0.0285 (5)	
H10A	0.6391	0.6877	0.3846	0.034*	
C11A	0.6488 (3)	0.8271 (2)	0.33405 (11)	0.0296 (5)	
H11A	0.5953	0.7704	0.3013	0.036*	
C12A	0.7001 (3)	0.9604 (2)	0.33048 (11)	0.0288 (5)	
C13A	0.7832 (3)	1.0403 (2)	0.38008 (11)	0.0301 (5)	
H13A	0.8232	1.1303	0.3792	0.036*	
C14A	0.8068 (3)	0.9889 (2)	0.42997 (11)	0.0285 (5)	
H14A	0.8625	1.0454	0.4626	0.034*	
N15A	0.6672 (3)	1.0079 (2)	0.28070 (9)	0.0358 (5)	
C16A	0.5310 (3)	0.9287 (3)	0.24208 (12)	0.0367 (6)	
H16A	0.4798	0.9840	0.2287	0.044*	
H16B	0.4612	0.8650	0.2641	0.044*	
C17A	0.5583 (3)	0.8580 (3)	0.18967 (11)	0.0365 (6)	
H17A	0.6124	0.9210	0.1637	0.044*	
H17B	0.6238	0.8135	0.2022	0.044*	
C18A	0.4126 (3)	0.7616 (3)	0.15729 (12)	0.0426 (7)	
H18A	0.3463	0.8061	0.1457	0.051*	
H18B	0.3598	0.6979	0.1832	0.051*	
C19A	0.4365 (3)	0.6919 (3)	0.10420 (13)	0.0492 (7)	
H19A	0.4856	0.7553	0.0776	0.059*	
H19B	0.5061	0.6503	0.1156	0.059*	
C20A	0.2913 (4)	0.5916 (4)	0.07301 (16)	0.0611 (9)	
H20A	0.2211	0.6328	0.0618	0.073*	
H20B	0.2426	0.5275	0.0994	0.073*	
C21A	0.3172 (6)	0.5242 (5)	0.0199 (2)	0.0898 (14)	
H21A	0.3559	0.5857	−0.0080	0.135*	
H21B	0.2216	0.4562	0.0032	0.135*	
H21C	0.3909	0.4871	0.0304	0.135*	
C22A	0.7282 (4)	1.1465 (3)	0.27664 (12)	0.0441 (7)	
H22A	0.8241	1.1864	0.3023	0.053*	
H22B	0.6562	1.1809	0.2905	0.053*	
C23A	0.7578 (3)	1.1851 (3)	0.21672 (13)	0.0458 (7)	
H23A	0.6626	1.1428	0.1908	0.055*	
H23B	0.7865	1.2783	0.2174	0.055*	
C24A	0.8803 (3)	1.1523 (3)	0.19208 (13)	0.0414 (6)	
H24A	0.8647	1.0635	0.1987	0.050*	
H24B	0.9802	1.2094	0.2121	0.050*	
C25A	0.8794 (4)	1.1664 (4)	0.12703 (16)	0.0613 (9)	
H25A	0.9547	1.1366	0.1125	0.074*	
H25B	0.7788	1.1096	0.1076	0.074*	
C26A	0.9133 (5)	1.2980 (4)	0.11120 (16)	0.0713 (11)	
H26A	1.0138	1.3557	0.1305	0.086*	
H26B	0.8374	1.3280	0.1249	0.086*	
C27A	0.9124 (7)	1.3054 (6)	0.04722 (19)	0.1085 (18)	
H27A	0.9906	1.2796	0.0337	0.163*	
H27B	0.9326	1.3932	0.0387	0.163*	
H27C	0.8133	1.2482	0.0279	0.163*	
N28A	0.7893 (2)	0.4731 (2)	0.70886 (10)	0.0372 (5)	
O29A	0.8486 (3)	0.5447 (2)	0.75293 (9)	0.0525 (6)	
O30A	0.7460 (3)	0.3564 (2)	0.70650 (10)	0.0582 (6)	
C1B	0.7306 (3)	0.1556 (2)	0.55379 (10)	0.0259 (5)	
C2B	0.8781 (3)	0.1709 (2)	0.57579 (11)	0.0295 (5)	
H2B	0.9621	0.2248	0.5585	0.035*	
C3B	0.9028 (3)	0.1091 (2)	0.62198 (11)	0.0299 (5)	
H3B	1.0025	0.1198	0.6361	0.036*	
C4B	0.7792 (3)	0.0308 (2)	0.64740 (10)	0.0284 (5)	
C5B	0.6329 (3)	0.0132 (2)	0.62734 (11)	0.0324 (5)	
H5B	0.5497	−0.0404	0.6451	0.039*	
C6B	0.6098 (3)	0.0750 (2)	0.58092 (11)	0.0311 (5)	
H6B	0.5095	0.0626	0.5669	0.037*	
C7B	0.6979 (3)	0.2171 (2)	0.50471 (10)	0.0283 (5)	
H7B	0.5943	0.1947	0.4918	0.034*	
C8B	0.7989 (3)	0.3022 (2)	0.47587 (10)	0.0281 (5)	
H8B	0.9025	0.3254	0.4891	0.034*	
C9B	0.7659 (3)	0.3625 (2)	0.42645 (10)	0.0267 (5)	
C10B	0.8844 (3)	0.4518 (2)	0.40158 (11)	0.0328 (5)	
H10B	0.9853	0.4735	0.4182	0.039*	
C11B	0.8600 (3)	0.5092 (3)	0.35394 (11)	0.0352 (6)	
H11B	0.9441	0.5697	0.3391	0.042*	
C12B	0.7125 (3)	0.4798 (2)	0.32682 (11)	0.0306 (5)	
C13B	0.5924 (3)	0.3903 (2)	0.35199 (11)	0.0320 (5)	
H13B	0.4914	0.3676	0.3353	0.038*	
C14B	0.6190 (3)	0.3355 (2)	0.40022 (11)	0.0301 (5)	
H14B	0.5352	0.2775	0.4162	0.036*	
N15B	0.6857 (3)	0.5360 (2)	0.27927 (10)	0.0399 (5)	
C16B	0.8077 (4)	0.6393 (3)	0.25697 (14)	0.0484 (7)	
H16C	0.8825	0.6912	0.2893	0.058*	
H16D	0.7651	0.6953	0.2382	0.058*	
C17B	0.8870 (4)	0.5911 (3)	0.21473 (15)	0.0531 (8)	
H17C	0.8140	0.5416	0.1815	0.064*	
H17D	0.9291	0.5341	0.2329	0.064*	
C18B	1.0185 (4)	0.7074 (4)	0.19407 (18)	0.0707 (11)	
H18C	1.0846	0.7591	0.2284	0.085*	
H18D	1.0796	0.6728	0.1721	0.085*	
C19B	0.9767 (6)	0.7943 (5)	0.1586 (2)	0.0883 (15)	
H19C	0.9155	0.8296	0.1801	0.106*	0.72
H19D	1.0702	0.8666	0.1523	0.106*	0.72
H19E	0.8662	0.7658	0.1590	0.106*	0.28
H19F	1.0218	0.8781	0.1817	0.106*	0.28
C20B	0.8959 (6)	0.7375 (5)	0.1057 (3)	0.0666 (14)	0.72
H20C	0.7942	0.6760	0.1117	0.080*	0.72
H20D	0.9481	0.6893	0.0868	0.080*	0.72
C20C	1.0014 (17)	0.8248 (15)	0.0996 (6)	0.068 (4)	0.28
H20E	1.0229	0.7558	0.0799	0.081*	0.28
H20F	1.0935	0.9041	0.1012	0.081*	0.28
C21B	0.8777 (7)	0.8424 (5)	0.0632 (2)	0.1038 (18)	
H21D	0.8136	0.8818	0.0791	0.156*	0.72
H21E	0.8312	0.7990	0.0253	0.156*	0.72
H21F	0.9775	0.9085	0.0602	0.156*	0.72
H21G	0.8265	0.8825	0.0870	0.156*	0.28
H21H	0.8049	0.7592	0.0456	0.156*	0.28
H21I	0.9207	0.8971	0.0333	0.156*	0.28
C22B	0.5380 (3)	0.4863 (3)	0.24494 (12)	0.0399 (6)	
H22C	0.5326	0.5515	0.2200	0.048*	
H22D	0.4585	0.4712	0.2708	0.048*	
C23B	0.5053 (3)	0.3612 (3)	0.20748 (14)	0.0473 (7)	
H23C	0.5847	0.3766	0.1816	0.057*	
H23D	0.5116	0.2963	0.2325	0.057*	
C24B	0.3527 (4)	0.3085 (4)	0.17207 (16)	0.0614 (9)	
H24C	0.3462	0.3727	0.1467	0.074*	
H24D	0.2729	0.2928	0.1978	0.074*	
C25B	0.3234 (5)	0.1819 (4)	0.13500 (18)	0.0762 (12)	
H25C	0.3402	0.1214	0.1604	0.091*	
H25D	0.2157	0.1442	0.1189	0.091*	
C26B	0.4170 (5)	0.1923 (5)	0.0872 (2)	0.0840 (13)	
H26C	0.5251	0.2268	0.1026	0.101*	
H26D	0.4019	0.2527	0.0613	0.101*	
C27B	0.3756 (7)	0.0624 (5)	0.0532 (2)	0.1055 (18)	
H27D	0.3901	0.0024	0.0788	0.158*	
H27E	0.4406	0.0721	0.0226	0.158*	
H27F	0.2697	0.0297	0.0366	0.158*	
N28B	0.8047 (3)	−0.0349 (2)	0.69623 (10)	0.0383 (5)	
O29B	0.9345 (3)	−0.0210 (2)	0.71236 (10)	0.0575 (6)	
O30B	0.6945 (3)	−0.1038 (2)	0.71804 (10)	0.0596 (6)	

### Atomic displacement parameters (Å^2^)

**Table:**

	*U*^11^	*U*^22^	*U*^33^	*U*^12^	*U*^13^	*U*^23^
C1A	0.0187 (10)	0.0273 (12)	0.0363 (13)	0.0110 (9)	0.0049 (9)	0.0032 (10)
C2A	0.0235 (11)	0.0274 (12)	0.0366 (13)	0.0103 (10)	0.0048 (9)	−0.0006 (10)
C3A	0.0227 (11)	0.0271 (12)	0.0420 (14)	0.0112 (10)	0.0078 (10)	0.0051 (10)
C4A	0.0209 (11)	0.0343 (13)	0.0363 (13)	0.0157 (10)	0.0081 (9)	0.0057 (10)
C5A	0.0281 (12)	0.0386 (14)	0.0339 (13)	0.0151 (11)	0.0007 (10)	−0.0012 (11)
C6A	0.0284 (12)	0.0262 (12)	0.0402 (14)	0.0114 (10)	0.0028 (10)	0.0016 (10)
C7A	0.0228 (11)	0.0276 (12)	0.0358 (13)	0.0100 (10)	0.0020 (9)	0.0010 (10)
C8A	0.0197 (11)	0.0283 (12)	0.0359 (13)	0.0096 (9)	0.0038 (9)	0.0011 (10)
C9A	0.0170 (10)	0.0252 (11)	0.0365 (13)	0.0103 (9)	0.0077 (9)	0.0046 (9)
C10A	0.0229 (11)	0.0223 (11)	0.0405 (13)	0.0083 (9)	0.0069 (10)	0.0036 (10)
C11A	0.0280 (12)	0.0280 (12)	0.0327 (12)	0.0105 (10)	0.0058 (10)	0.0015 (10)
C12A	0.0249 (11)	0.0306 (12)	0.0357 (13)	0.0139 (10)	0.0111 (10)	0.0070 (10)
C13A	0.0278 (12)	0.0228 (11)	0.0410 (14)	0.0101 (10)	0.0098 (10)	0.0027 (10)
C14A	0.0222 (11)	0.0256 (12)	0.0381 (13)	0.0097 (9)	0.0058 (9)	0.0010 (10)
N15A	0.0406 (12)	0.0324 (11)	0.0357 (12)	0.0147 (10)	0.0066 (9)	0.0077 (9)
C16A	0.0326 (13)	0.0430 (15)	0.0418 (15)	0.0208 (12)	0.0088 (11)	0.0118 (12)
C17A	0.0317 (13)	0.0415 (15)	0.0393 (14)	0.0165 (12)	0.0062 (11)	0.0076 (11)
C18A	0.0302 (14)	0.0505 (17)	0.0442 (16)	0.0120 (12)	0.0047 (11)	0.0087 (13)
C19A	0.0385 (16)	0.0561 (19)	0.0482 (17)	0.0134 (14)	0.0042 (13)	0.0054 (14)
C20A	0.054 (2)	0.060 (2)	0.060 (2)	0.0147 (17)	−0.0010 (16)	−0.0039 (17)
C21A	0.085 (3)	0.087 (3)	0.084 (3)	0.026 (3)	0.005 (2)	−0.026 (3)
C22A	0.0618 (19)	0.0332 (14)	0.0434 (16)	0.0226 (14)	0.0147 (14)	0.0107 (12)
C23A	0.0422 (16)	0.0447 (16)	0.0552 (18)	0.0197 (13)	0.0109 (13)	0.0155 (14)
C24A	0.0304 (14)	0.0432 (16)	0.0539 (17)	0.0160 (12)	0.0098 (12)	0.0130 (13)
C25A	0.0460 (19)	0.070 (2)	0.071 (2)	0.0246 (17)	0.0136 (16)	0.0029 (19)
C26A	0.063 (2)	0.080 (3)	0.063 (2)	0.015 (2)	0.0167 (19)	0.021 (2)
C27A	0.129 (5)	0.131 (5)	0.060 (3)	0.040 (4)	0.014 (3)	0.036 (3)
N28A	0.0298 (11)	0.0476 (14)	0.0411 (13)	0.0211 (10)	0.0074 (9)	0.0115 (11)
O29A	0.0680 (15)	0.0632 (14)	0.0372 (11)	0.0392 (12)	−0.0007 (10)	0.0037 (10)
O30A	0.0649 (15)	0.0431 (12)	0.0606 (14)	0.0135 (11)	0.0012 (11)	0.0210 (11)
C1B	0.0266 (12)	0.0211 (11)	0.0322 (12)	0.0114 (9)	0.0067 (9)	0.0009 (9)
C2B	0.0210 (11)	0.0297 (12)	0.0373 (13)	0.0083 (10)	0.0081 (9)	0.0035 (10)
C3B	0.0225 (11)	0.0302 (12)	0.0366 (13)	0.0104 (10)	0.0031 (9)	0.0012 (10)
C4B	0.0304 (12)	0.0261 (12)	0.0326 (12)	0.0142 (10)	0.0072 (10)	0.0049 (9)
C5B	0.0252 (12)	0.0308 (13)	0.0426 (14)	0.0104 (10)	0.0120 (10)	0.0074 (11)
C6B	0.0203 (11)	0.0304 (12)	0.0448 (14)	0.0111 (10)	0.0076 (10)	0.0064 (11)
C7B	0.0250 (11)	0.0267 (12)	0.0361 (13)	0.0132 (10)	0.0048 (9)	0.0010 (10)
C8B	0.0244 (11)	0.0279 (12)	0.0345 (13)	0.0130 (10)	0.0041 (9)	0.0006 (10)
C9B	0.0251 (11)	0.0252 (11)	0.0332 (12)	0.0128 (9)	0.0075 (9)	0.0018 (9)
C10B	0.0232 (12)	0.0349 (13)	0.0393 (14)	0.0098 (10)	0.0051 (10)	0.0044 (11)
C11B	0.0258 (12)	0.0352 (14)	0.0424 (15)	0.0081 (11)	0.0083 (11)	0.0095 (11)
C12B	0.0304 (13)	0.0292 (12)	0.0369 (13)	0.0154 (10)	0.0084 (10)	0.0072 (10)
C13B	0.0246 (12)	0.0351 (13)	0.0404 (14)	0.0154 (10)	0.0065 (10)	0.0059 (11)
C14B	0.0258 (12)	0.0282 (12)	0.0376 (13)	0.0101 (10)	0.0106 (10)	0.0070 (10)
N15B	0.0327 (12)	0.0424 (13)	0.0471 (13)	0.0154 (10)	0.0067 (10)	0.0167 (11)
C16B	0.0522 (18)	0.0490 (18)	0.0496 (17)	0.0233 (15)	0.0124 (14)	0.0159 (14)
C17B	0.0476 (18)	0.058 (2)	0.063 (2)	0.0310 (16)	0.0076 (15)	0.0102 (16)
C18B	0.051 (2)	0.092 (3)	0.076 (3)	0.028 (2)	0.0311 (19)	0.031 (2)
C19B	0.101 (4)	0.085 (3)	0.100 (4)	0.047 (3)	0.053 (3)	0.036 (3)
C20B	0.052 (3)	0.050 (3)	0.098 (4)	0.025 (2)	−0.002 (3)	−0.010 (3)
C20C	0.072 (9)	0.079 (10)	0.060 (8)	0.034 (8)	0.011 (7)	0.029 (7)
C21B	0.128 (5)	0.109 (4)	0.095 (4)	0.077 (4)	−0.021 (3)	−0.002 (3)
C22B	0.0383 (15)	0.0479 (16)	0.0432 (15)	0.0257 (13)	0.0066 (12)	0.0127 (13)
C23B	0.0376 (16)	0.0514 (18)	0.0565 (19)	0.0209 (14)	0.0056 (13)	0.0078 (14)
C24B	0.0423 (18)	0.075 (2)	0.071 (2)	0.0289 (17)	0.0038 (16)	−0.0003 (19)
C25B	0.053 (2)	0.089 (3)	0.074 (3)	0.020 (2)	−0.0077 (19)	−0.009 (2)
C26B	0.074 (3)	0.094 (3)	0.080 (3)	0.031 (3)	−0.003 (2)	0.007 (3)
C27B	0.145 (5)	0.113 (4)	0.078 (3)	0.078 (4)	0.000 (3)	−0.013 (3)
N28B	0.0439 (13)	0.0387 (13)	0.0393 (12)	0.0223 (11)	0.0091 (10)	0.0095 (10)
O29B	0.0466 (13)	0.0743 (16)	0.0606 (14)	0.0321 (12)	0.0014 (10)	0.0250 (12)
O30B	0.0543 (14)	0.0668 (15)	0.0651 (15)	0.0247 (12)	0.0219 (11)	0.0365 (12)

### Geometric parameters (Å, °)

**Table:**

C1A—C2A	1.401 (3)	C3B—C4B	1.394 (3)
C1A—C6A	1.411 (3)	C3B—H3B	0.9500
C1A—C7A	1.466 (3)	C4B—C5B	1.385 (3)
C2A—C3A	1.390 (3)	C4B—N28B	1.465 (3)
C2A—H2A	0.9500	C5B—C6B	1.384 (4)
C3A—C4A	1.384 (4)	C5B—H5B	0.9500
C3A—H3A	0.9500	C6B—H6B	0.9500
C4A—C5A	1.394 (4)	C7B—C8B	1.347 (3)
C4A—N28A	1.466 (3)	C7B—H7B	0.9500
C5A—C6A	1.390 (4)	C8B—C9B	1.461 (3)
C5A—H5A	0.9500	C8B—H8B	0.9500
C6A—H6A	0.9500	C9B—C10B	1.406 (3)
C7A—C8A	1.342 (3)	C9B—C14B	1.408 (3)
C7A—H7A	0.9500	C10B—C11B	1.381 (4)
C8A—C9A	1.460 (3)	C10B—H10B	0.9500
C8A—H8A	0.9500	C11B—C12B	1.416 (4)
C9A—C14A	1.403 (3)	C11B—H11B	0.9500
C9A—C10A	1.414 (3)	C12B—N15B	1.378 (3)
C10A—C11A	1.382 (3)	C12B—C13B	1.419 (3)
C10A—H10A	0.9500	C13B—C14B	1.382 (3)
C11A—C12A	1.417 (3)	C13B—H13B	0.9500
C11A—H11A	0.9500	C14B—H14B	0.9500
C12A—N15A	1.385 (3)	N15B—C22B	1.463 (4)
C12A—C13A	1.414 (4)	N15B—C16B	1.469 (4)
C13A—C14A	1.386 (3)	C16B—C17B	1.508 (4)
C13A—H13A	0.9500	C16B—H16C	0.9900
C14A—H14A	0.9500	C16B—H16D	0.9900
N15A—C16A	1.471 (3)	C17B—C18B	1.578 (5)
N15A—C22A	1.471 (3)	C17B—H17C	0.9900
C16A—C17A	1.535 (4)	C17B—H17D	0.9900
C16A—H16A	0.9900	C18B—C19B	1.482 (6)
C16A—H16B	0.9900	C18B—H18C	0.9900
C17A—C18A	1.518 (4)	C18B—H18D	0.9900
C17A—H17A	0.9900	C19B—C20B	1.393 (7)
C17A—H17B	0.9900	C19B—C20C	1.472 (13)
C18A—C19A	1.527 (4)	C19B—H19C	0.9900
C18A—H18A	0.9900	C19B—H19D	0.9900
C18A—H18B	0.9900	C19B—H19E	0.9900
C19A—C20A	1.520 (4)	C19B—H19F	0.9900
C19A—H19A	0.9900	C20B—C21B	1.655 (7)
C19A—H19B	0.9900	C20B—H20C	0.9900
C20A—C21A	1.523 (5)	C20B—H20D	0.9900
C20A—H20A	0.9900	C20C—C21B	1.495 (14)
C20A—H20B	0.9900	C20C—H20E	0.9900
C21A—H21A	0.9800	C20C—H20F	0.9900
C21A—H21B	0.9800	C21B—H21D	0.9800
C21A—H21C	0.9800	C21B—H21E	0.9800
C22A—C23A	1.523 (4)	C21B—H21F	0.9800
C22A—H22A	0.9900	C21B—H21G	0.9800
C22A—H22B	0.9900	C21B—H21H	0.9800
C23A—C24A	1.520 (4)	C21B—H21I	0.9800
C23A—H23A	0.9900	C22B—C23B	1.543 (4)
C23A—H23B	0.9900	C22B—H22C	0.9900
C24A—C25A	1.553 (5)	C22B—H22D	0.9900
C24A—H24A	0.9900	C23B—C24B	1.511 (4)
C24A—H24B	0.9900	C23B—H23C	0.9900
C25A—C26A	1.487 (5)	C23B—H23D	0.9900
C25A—H25A	0.9900	C24B—C25B	1.559 (5)
C25A—H25B	0.9900	C24B—H24C	0.9900
C26A—C27A	1.517 (5)	C24B—H24D	0.9900
C26A—H26A	0.9900	C25B—C26B	1.487 (6)
C26A—H26B	0.9900	C25B—H25C	0.9900
C27A—H27A	0.9800	C25B—H25D	0.9900
C27A—H27B	0.9800	C26B—C27B	1.534 (6)
C27A—H27C	0.9800	C26B—H26C	0.9900
N28A—O30A	1.229 (3)	C26B—H26D	0.9900
N28A—O29A	1.232 (3)	C27B—H27D	0.9800
C1B—C6B	1.408 (3)	C27B—H27E	0.9800
C1B—C2B	1.415 (3)	C27B—H27F	0.9800
C1B—C7B	1.462 (3)	N28B—O29B	1.225 (3)
C2B—C3B	1.385 (3)	N28B—O30B	1.229 (3)
C2B—H2B	0.9500		
			
C2A—C1A—C6A	117.9 (2)	C6B—C5B—H5B	120.6
C2A—C1A—C7A	118.5 (2)	C4B—C5B—H5B	120.6
C6A—C1A—C7A	123.6 (2)	C5B—C6B—C1B	122.0 (2)
C3A—C2A—C1A	121.6 (2)	C5B—C6B—H6B	119.0
C3A—C2A—H2A	119.2	C1B—C6B—H6B	119.0
C1A—C2A—H2A	119.2	C8B—C7B—C1B	127.0 (2)
C4A—C3A—C2A	118.8 (2)	C8B—C7B—H7B	116.5
C4A—C3A—H3A	120.6	C1B—C7B—H7B	116.5
C2A—C3A—H3A	120.6	C7B—C8B—C9B	126.9 (2)
C3A—C4A—C5A	121.6 (2)	C7B—C8B—H8B	116.6
C3A—C4A—N28A	119.0 (2)	C9B—C8B—H8B	116.6
C5A—C4A—N28A	119.4 (2)	C10B—C9B—C14B	115.9 (2)
C6A—C5A—C4A	118.9 (2)	C10B—C9B—C8B	120.1 (2)
C6A—C5A—H5A	120.5	C14B—C9B—C8B	123.9 (2)
C4A—C5A—H5A	120.5	C11B—C10B—C9B	122.6 (2)
C5A—C6A—C1A	121.0 (2)	C11B—C10B—H10B	118.7
C5A—C6A—H6A	119.5	C9B—C10B—H10B	118.7
C1A—C6A—H6A	119.5	C10B—C11B—C12B	121.4 (2)
C8A—C7A—C1A	127.0 (2)	C10B—C11B—H11B	119.3
C8A—C7A—H7A	116.5	C12B—C11B—H11B	119.3
C1A—C7A—H7A	116.5	N15B—C12B—C11B	122.4 (2)
C7A—C8A—C9A	126.7 (2)	N15B—C12B—C13B	121.5 (2)
C7A—C8A—H8A	116.6	C11B—C12B—C13B	116.1 (2)
C9A—C8A—H8A	116.6	C14B—C13B—C12B	121.6 (2)
C14A—C9A—C10A	116.1 (2)	C14B—C13B—H13B	119.2
C14A—C9A—C8A	120.4 (2)	C12B—C13B—H13B	119.2
C10A—C9A—C8A	123.4 (2)	C13B—C14B—C9B	122.3 (2)
C11A—C10A—C9A	121.8 (2)	C13B—C14B—H14B	118.8
C11A—C10A—H10A	119.1	C9B—C14B—H14B	118.8
C9A—C10A—H10A	119.1	C12B—N15B—C22B	121.4 (2)
C10A—C11A—C12A	121.7 (2)	C12B—N15B—C16B	121.7 (2)
C10A—C11A—H11A	119.2	C22B—N15B—C16B	116.6 (2)
C12A—C11A—H11A	119.2	N15B—C16B—C17B	113.0 (3)
N15A—C12A—C13A	122.6 (2)	N15B—C16B—H16C	109.0
N15A—C12A—C11A	120.7 (2)	C17B—C16B—H16C	109.0
C13A—C12A—C11A	116.7 (2)	N15B—C16B—H16D	109.0
C14A—C13A—C12A	120.8 (2)	C17B—C16B—H16D	109.0
C14A—C13A—H13A	119.6	H16C—C16B—H16D	107.8
C12A—C13A—H13A	119.6	C16B—C17B—C18B	109.8 (3)
C13A—C14A—C9A	122.8 (2)	C16B—C17B—H17C	109.7
C13A—C14A—H14A	118.6	C18B—C17B—H17C	109.7
C9A—C14A—H14A	118.6	C16B—C17B—H17D	109.7
C12A—N15A—C16A	118.6 (2)	C18B—C17B—H17D	109.7
C12A—N15A—C22A	120.0 (2)	H17C—C17B—H17D	108.2
C16A—N15A—C22A	117.4 (2)	C19B—C18B—C17B	118.0 (3)
N15A—C16A—C17A	115.4 (2)	C19B—C18B—H18C	107.8
N15A—C16A—H16A	108.4	C17B—C18B—H18C	107.8
C17A—C16A—H16A	108.4	C19B—C18B—H18D	107.8
N15A—C16A—H16B	108.4	C17B—C18B—H18D	107.8
C17A—C16A—H16B	108.4	H18C—C18B—H18D	107.1
H16A—C16A—H16B	107.5	C20B—C19B—C18B	114.5 (5)
C18A—C17A—C16A	112.6 (2)	C20C—C19B—C18B	131.5 (7)
C18A—C17A—H17A	109.1	C20B—C19B—H19C	108.6
C16A—C17A—H17A	109.1	C20C—C19B—H19C	119.7
C18A—C17A—H17B	109.1	C18B—C19B—H19C	108.6
C16A—C17A—H17B	109.1	C20B—C19B—H19D	108.6
H17A—C17A—H17B	107.8	C18B—C19B—H19D	108.6
C17A—C18A—C19A	113.6 (2)	H19C—C19B—H19D	107.6
C17A—C18A—H18A	108.8	C20C—C19B—H19E	104.4
C19A—C18A—H18A	108.8	C18B—C19B—H19E	104.4
C17A—C18A—H18B	108.8	C20C—C19B—H19F	104.4
C19A—C18A—H18B	108.8	C18B—C19B—H19F	104.4
H18A—C18A—H18B	107.7	H19E—C19B—H19F	105.6
C20A—C19A—C18A	113.7 (3)	C19B—C20B—C21B	112.8 (4)
C20A—C19A—H19A	108.8	C19B—C20B—H20C	109.0
C18A—C19A—H19A	108.8	C21B—C20B—H20C	109.0
C20A—C19A—H19B	108.8	C19B—C20B—H20D	109.0
C18A—C19A—H19B	108.8	C21B—C20B—H20D	109.0
H19A—C19A—H19B	107.7	H20C—C20B—H20D	107.8
C19A—C20A—C21A	112.9 (3)	C19B—C20C—C21B	117.9 (10)
C19A—C20A—H20A	109.0	C19B—C20C—H20E	107.8
C21A—C20A—H20A	109.0	C21B—C20C—H20E	107.8
C19A—C20A—H20B	109.0	C19B—C20C—H20F	107.8
C21A—C20A—H20B	109.0	C21B—C20C—H20F	107.8
H20A—C20A—H20B	107.8	H20E—C20C—H20F	107.2
C20A—C21A—H21A	109.5	C20C—C21B—H21D	121.9
C20A—C21A—H21B	109.5	C20B—C21B—H21D	109.5
H21A—C21A—H21B	109.5	C20C—C21B—H21E	126.7
C20A—C21A—H21C	109.5	C20B—C21B—H21E	109.5
H21A—C21A—H21C	109.5	H21D—C21B—H21E	109.5
H21B—C21A—H21C	109.5	C20B—C21B—H21F	109.5
N15A—C22A—C23A	114.5 (2)	H21D—C21B—H21F	109.5
N15A—C22A—H22A	108.6	H21E—C21B—H21F	109.5
C23A—C22A—H22A	108.6	C20C—C21B—H21G	109.5
N15A—C22A—H22B	108.6	C20B—C21B—H21G	100.2
C23A—C22A—H22B	108.6	H21E—C21B—H21G	121.3
H22A—C22A—H22B	107.6	H21F—C21B—H21G	106.3
C24A—C23A—C22A	114.9 (2)	C20C—C21B—H21H	109.5
C24A—C23A—H23A	108.5	H21D—C21B—H21H	103.3
C22A—C23A—H23A	108.5	H21G—C21B—H21H	109.5
C24A—C23A—H23B	108.5	C20C—C21B—H21I	109.5
C22A—C23A—H23B	108.5	H21D—C21B—H21I	102.6
H23A—C23A—H23B	107.5	H21G—C21B—H21I	109.5
C23A—C24A—C25A	111.7 (2)	H21H—C21B—H21I	109.5
C23A—C24A—H24A	109.3	N15B—C22B—C23B	113.5 (2)
C25A—C24A—H24A	109.3	N15B—C22B—H22C	108.9
C23A—C24A—H24B	109.3	C23B—C22B—H22C	108.9
C25A—C24A—H24B	109.3	N15B—C22B—H22D	108.9
H24A—C24A—H24B	107.9	C23B—C22B—H22D	108.9
C26A—C25A—C24A	115.0 (3)	H22C—C22B—H22D	107.7
C26A—C25A—H25A	108.5	C24B—C23B—C22B	113.9 (3)
C24A—C25A—H25A	108.5	C24B—C23B—H23C	108.8
C26A—C25A—H25B	108.5	C22B—C23B—H23C	108.8
C24A—C25A—H25B	108.5	C24B—C23B—H23D	108.8
H25A—C25A—H25B	107.5	C22B—C23B—H23D	108.8
C25A—C26A—C27A	112.3 (4)	H23C—C23B—H23D	107.7
C25A—C26A—H26A	109.1	C23B—C24B—C25B	112.5 (3)
C27A—C26A—H26A	109.1	C23B—C24B—H24C	109.1
C25A—C26A—H26B	109.1	C25B—C24B—H24C	109.1
C27A—C26A—H26B	109.1	C23B—C24B—H24D	109.1
H26A—C26A—H26B	107.9	C25B—C24B—H24D	109.1
C26A—C27A—H27A	109.5	H24C—C24B—H24D	107.8
C26A—C27A—H27B	109.5	C26B—C25B—C24B	116.3 (4)
H27A—C27A—H27B	109.5	C26B—C25B—H25C	108.2
C26A—C27A—H27C	109.5	C24B—C25B—H25C	108.2
H27A—C27A—H27C	109.5	C26B—C25B—H25D	108.2
H27B—C27A—H27C	109.5	C24B—C25B—H25D	108.2
O30A—N28A—O29A	123.6 (2)	H25C—C25B—H25D	107.4
O30A—N28A—C4A	117.8 (2)	C25B—C26B—C27B	111.4 (4)
O29A—N28A—C4A	118.5 (2)	C25B—C26B—H26C	109.3
C6B—C1B—C2B	117.2 (2)	C27B—C26B—H26C	109.3
C6B—C1B—C7B	119.1 (2)	C25B—C26B—H26D	109.3
C2B—C1B—C7B	123.7 (2)	C27B—C26B—H26D	109.3
C3B—C2B—C1B	121.5 (2)	H26C—C26B—H26D	108.0
C3B—C2B—H2B	119.3	C26B—C27B—H27D	109.5
C1B—C2B—H2B	119.3	C26B—C27B—H27E	109.5
C2B—C3B—C4B	119.0 (2)	H27D—C27B—H27E	109.5
C2B—C3B—H3B	120.5	C26B—C27B—H27F	109.5
C4B—C3B—H3B	120.5	H27D—C27B—H27F	109.5
C5B—C4B—C3B	121.6 (2)	H27E—C27B—H27F	109.5
C5B—C4B—N28B	119.3 (2)	O29B—N28B—O30B	123.2 (2)
C3B—C4B—N28B	119.2 (2)	O29B—N28B—C4B	118.4 (2)
C6B—C5B—C4B	118.9 (2)	O30B—N28B—C4B	118.4 (2)
			
C6A—C1A—C2A—C3A	−1.3 (3)	C2B—C3B—C4B—C5B	0.4 (4)
C7A—C1A—C2A—C3A	178.5 (2)	C2B—C3B—C4B—N28B	179.9 (2)
C1A—C2A—C3A—C4A	0.8 (3)	C3B—C4B—C5B—C6B	0.0 (4)
C2A—C3A—C4A—C5A	0.5 (3)	N28B—C4B—C5B—C6B	−179.5 (2)
C2A—C3A—C4A—N28A	−178.4 (2)	C4B—C5B—C6B—C1B	−0.4 (4)
C3A—C4A—C5A—C6A	−1.4 (3)	C2B—C1B—C6B—C5B	0.3 (4)
N28A—C4A—C5A—C6A	177.5 (2)	C7B—C1B—C6B—C5B	179.6 (2)
C4A—C5A—C6A—C1A	0.9 (4)	C6B—C1B—C7B—C8B	175.9 (2)
C2A—C1A—C6A—C5A	0.4 (3)	C2B—C1B—C7B—C8B	−4.8 (4)
C7A—C1A—C6A—C5A	−179.4 (2)	C1B—C7B—C8B—C9B	179.3 (2)
C2A—C1A—C7A—C8A	177.2 (2)	C7B—C8B—C9B—C10B	178.8 (2)
C6A—C1A—C7A—C8A	−3.0 (4)	C7B—C8B—C9B—C14B	−2.4 (4)
C1A—C7A—C8A—C9A	−178.2 (2)	C14B—C9B—C10B—C11B	−0.7 (4)
C7A—C8A—C9A—C14A	−179.1 (2)	C8B—C9B—C10B—C11B	178.3 (2)
C7A—C8A—C9A—C10A	2.7 (4)	C9B—C10B—C11B—C12B	−0.7 (4)
C14A—C9A—C10A—C11A	−2.7 (3)	C10B—C11B—C12B—N15B	180.0 (2)
C8A—C9A—C10A—C11A	175.6 (2)	C10B—C11B—C12B—C13B	1.1 (4)
C9A—C10A—C11A—C12A	0.7 (4)	N15B—C12B—C13B—C14B	−178.9 (2)
C10A—C11A—C12A—N15A	−177.1 (2)	C11B—C12B—C13B—C14B	0.0 (4)
C10A—C11A—C12A—C13A	1.9 (3)	C12B—C13B—C14B—C9B	−1.5 (4)
N15A—C12A—C13A—C14A	176.5 (2)	C10B—C9B—C14B—C13B	1.8 (4)
C11A—C12A—C13A—C14A	−2.5 (3)	C8B—C9B—C14B—C13B	−177.1 (2)
C12A—C13A—C14A—C9A	0.4 (4)	C11B—C12B—N15B—C22B	167.1 (2)
C10A—C9A—C14A—C13A	2.1 (3)	C13B—C12B—N15B—C22B	−14.0 (4)
C8A—C9A—C14A—C13A	−176.1 (2)	C11B—C12B—N15B—C16B	−6.0 (4)
C13A—C12A—N15A—C16A	−152.7 (2)	C13B—C12B—N15B—C16B	172.9 (3)
C11A—C12A—N15A—C16A	26.2 (3)	C12B—N15B—C16B—C17B	88.2 (3)
C13A—C12A—N15A—C22A	4.3 (4)	C22B—N15B—C16B—C17B	−85.2 (3)
C11A—C12A—N15A—C22A	−176.7 (2)	N15B—C16B—C17B—C18B	−178.6 (3)
C12A—N15A—C16A—C17A	−100.9 (3)	C16B—C17B—C18B—C19B	−67.8 (5)
C22A—N15A—C16A—C17A	101.5 (3)	C17B—C18B—C19B—C20B	−63.9 (6)
N15A—C16A—C17A—C18A	170.3 (2)	C17B—C18B—C19B—C20C	−117.3 (10)
C16A—C17A—C18A—C19A	178.8 (2)	C20C—C19B—C20B—C21B	−45.2 (9)
C17A—C18A—C19A—C20A	177.7 (3)	C18B—C19B—C20B—C21B	−170.0 (4)
C18A—C19A—C20A—C21A	179.5 (3)	C20B—C19B—C20C—C21B	55.2 (10)
C12A—N15A—C22A—C23A	149.4 (2)	C18B—C19B—C20C—C21B	141.3 (8)
C16A—N15A—C22A—C23A	−53.3 (3)	C19B—C20C—C21B—C20B	−48.7 (8)
N15A—C22A—C23A—C24A	−65.1 (4)	C19B—C20B—C21B—C20C	49.5 (9)
C22A—C23A—C24A—C25A	167.3 (3)	C12B—N15B—C22B—C23B	−74.1 (3)
C23A—C24A—C25A—C26A	63.3 (4)	C16B—N15B—C22B—C23B	99.4 (3)
C24A—C25A—C26A—C27A	179.4 (3)	N15B—C22B—C23B—C24B	179.6 (3)
C3A—C4A—N28A—O30A	4.3 (3)	C22B—C23B—C24B—C25B	−179.8 (3)
C5A—C4A—N28A—O30A	−174.7 (2)	C23B—C24B—C25B—C26B	−68.7 (5)
C3A—C4A—N28A—O29A	−176.5 (2)	C24B—C25B—C26B—C27B	−178.8 (4)
C5A—C4A—N28A—O29A	4.6 (3)	C5B—C4B—N28B—O29B	178.0 (2)
C6B—C1B—C2B—C3B	0.1 (3)	C3B—C4B—N28B—O29B	−1.6 (4)
C7B—C1B—C2B—C3B	−179.1 (2)	C5B—C4B—N28B—O30B	−0.2 (4)
C1B—C2B—C3B—C4B	−0.4 (4)	C3B—C4B—N28B—O30B	−179.8 (2)
